# Designing and development of agricultural rovers for vegetable harvesting and soil analysis

**DOI:** 10.1371/journal.pone.0304657

**Published:** 2024-06-21

**Authors:** Bristy Das, Tahmid Zarif Ul Hoq Sayor, Rubyat Jahan Nijhum, Mehnaz Tabassum Tishun, Taiyeb Hasan Sakib, Md. Ehsanul Karim, AFM Jamal Uddin, Aparna Islam, Abu S. M. Mohsin

**Affiliations:** 1 EEE Department, Nanotechnology, IoT and Applied Machine Learning Research Group, Brac University, Dhaka, Bangladesh; 2 Department of Horticulture, Sher-e-Bangla Agricultural University (SAU), Dhaka, Bangladesh; 3 Department of Mathematics and Natural Sciences, Biotechnology Program, Brac University, Dhaka, Bangladesh; University of Jeddah, SAUDI ARABIA

## Abstract

To address the growing demand for sustainable agriculture practices, new technologies to boost crop productivity and soil health must be developed. In this research, we propose designing and building an agricultural rover capable of autonomous vegetable harvesting and soil analysis utilizing cutting-edge deep learning algorithms (YOLOv5). The precision and recall score of the model was 0.8518% and 0.7624% respectively. The rover uses robotics, computer vision, and soil sensing technology to perform accurate and efficient agricultural tasks. We go over the rover’s hardware and software, as well as the soil analysis system and the tomato ripeness detection system using deep learning models. Field experiments indicate that this agricultural rover is effective and promising for improving crop management and soil monitoring in modern agriculture, hence achieving the UN’s SDG 2 Zero Hunger goals.

## Introduction

Global population growth poses a significant challenge for agriculture in fulfilling rising food demand. Precision agriculture and automation are crucial in meeting this problem by increasing agricultural productivity while saving resources. In this study, we propose an agricultural rover outfitted with advanced deep-learning algorithms to alter vegetable harvesting and soil analysis in farming practices.

In the recent era, precision agriculture has played an important role in maintaining future food security with less labor and energy and at the same time improving environmental management to ensure a productive agricultural output [1]. Many regions face labor shortages in agriculture due to changing demographics and migration to urban areas. Automation can assist in bridging this gap by reducing the requirement for human labor. While an initial investment in automation technologies may be required, long-term cost savings can be realized by cutting labor expenses and increasing farm output.

Sensor-enabled growth monitoring systems, drones, and satellite photography can provide real-time information about crop health, nutritional deficits, and growth rates. This data enables farmers to make better decisions about irrigation, fertilization, and pest management, resulting in increased crop growth and yields.

Mechanical harvesting systems improve profitability and efficiency in horticultural businesses, but often damage fruits, necessitating the development of efficient automated harvesting methods to maintain vegetable quality [2]. Crops are harvested at their peak nutritional content and flavor when chosen at the right time, resulting in a higher-quality product with a higher market value. Automation can help to improve agricultural sustainability by optimizing resource usage. Water waste can be reduced by using automated irrigation systems, which deliver water to crops based on their needs. Similarly, soil testing is crucial for modern agriculture to optimize production, protect the environment from the overuse of fertilizers, and save money and energy [3]. Automated systems create massive amounts of data, giving farmers vital insights for decision-making. For example, automated soil analysis provides detailed information about soil health and nutrient levels, allowing farmers to adjust their agricultural inputs.

Bangladesh is a thriving agricultural nation. Bangladesh’s economy and livelihood are mostly based on agriculture. She is self-sufficient in cereals and vegetables and fruits are largely produced locally, indicating there is still a need for yield increment in this area. Several factors are responsible for agricultural enhancements which will not only enhance the public’s economic situation but also improve the standard of living. Additionally, if the production rate increases it will create a great effect on import business and thus in the economy. The improvements in agriculture can be done in many ways by utilizing new innovations. However, our country is yet to completely embrace these innovative breakthroughs, particularly in agriculture. Even though agriculture is the backbone of our nation’s economy, most farmers are unaware of the latest developments in farming technology. Most farmers in our nation suffer from a lack of technological understanding of soil analysis and its significance. Agricultural robots are being used in several nations to improve life quality, make work easier, and raise crop yields. Technology has always had a significant impact on making life easier, faster, and more productive. Statistics from technologically sophisticated nations show that robotics and technology can dramatically impact agricultural growth. Technological innovations were responsible for a 15.7 percent increase in the overall performance of China, a 47.4 percent increase in overall performance in Japan, and an 84.2 percent increase in the United States between 1960 and 1980 [4]. On the other hand, Bangladeshi farmers are in a perilous position in this quickly changing industry. Again, despite the country’s high unemployment rate, many young people find agriculture uninteresting because it involves more effort and hard work. Farmers are having difficulty finding labor to perform harvesting operations these days. This is a severe problem since it causes agricultural harvesting to be delayed. Agriculture is tied to a slew of other societal issues and concerns because it has such a large economic impact on our country [5]. If robotics is involved, the younger generation will be encouraged to engage in this industry, and smart agriculture may even help Bangladesh reduce its unemployment rate.

In conclusion, automation plays a crucial role in modern agriculture, especially in harvesting, growth monitoring, and soil analysis. It enhances efficiency, reduces labor dependency, ensures precision, and contributes to sustainable agricultural practices. As the world’s population continues to grow, embracing automation in agriculture becomes increasingly necessary to meet global food demands while maintaining environmental sustainability.

## Background study

In this section, previous research efforts in the fields of precision agriculture, computer vision, and autonomous robotics are discussed. Notably, works related to vegetable detection and harvesting, soil analysis using sensors, and the integration of deep learning techniques in agricultural systems are highlighted.

There has been a study on a system that is developed for detecting and picking tomatoes in greenhouses where the main technology is the effective detection of ripe tomatoes against a complicated background [5]. The ripe tomato is segmented using the L*a*b* color image and K-means clustering. The shape-based method is used to eliminate noise and manage the conditions of tomato overlaying and shelter to obtain single-integrity ripened tomatoes. This paper proposes a ripe tomato extraction technique based on color clustering and mathematical morphology [5]. However, there are some drawbacks in this study. The ripe tomato is only taking up a small portion of the picture, resulting in incorrect cluster segmentation for the ripe tomato. To ensure that the correct ripe tomato is always included in the input images, the ripe tomato detective should be used. The study solely focuses on the detection process and does not provide a sufficient harvesting procedure or mechanical description for the system. The mechanical structural information regarding the harvesting system is obscure, therefore the notion of implementing this system in a regular field with unplanned arrangement remains uncertain. Furthermore, the system is only capable of recognizing and harvesting tomatoes; it cannot perform any other activities.

Again, in another study, there has been a grip-type end-effector developed for use in a picking robot, designed to harvest greenhouse-grown tomatoes [6]. The end-effector has been developed with four fingers that contain foam sponge pads inside to lessen bending and grabbing damage to the fruit. A fruit suction mechanism is in the middle of the end-effector, helping to fix the fruit there and improving holding capacity. The findings indicate that before selecting tomatoes, the end-effector should be turned three times about the fruit, first clockwise for 60°, then counterclockwise for 120°, until the fruit is 60° counterclockwise to the initial alignment. The findings indicate that before selecting tomatoes, the end-effector should be turned three times in reference to the fruit, first clockwise for 60°, then counterclockwise for 120°, until the fruit is 60° counterclockwise to the initial alignment [6]. Nevertheless, the system is designed to be implemented in a greenhouse environment only. The equipment displayed here cannot travel autonomously; instead, it follows a predetermined path to complete the tasks. This limits the device’s use in ordinary fields. The gadget is insufficient for all sectors and environments. Again, the device’s functions are limited to a single purpose only. Again, there will be some accuracy issues in the case of clustered tomato harvesting.

Moreover, in another study, Nitrogen (N), Phosphorus (P), and Potassium (K), NPK sensors will be used to detect the soil nutrients. The results identified by the sensor will be delivered in the form of analog signal data to NodeMCU, which will be processed and presented on the screen [7]. The NPK sensor tool’s job is to measure the nutrients in the soil for citrus seedlings, and the data will be communicated to Thingspeak online. This research focuses on developing a soil nutrient measurement tool that makes use of NPK soil sensors and Internet of Things (IoT) [7]. This work is useful for testing soil nutrients. Regardless of how, the technique indicated in the paper is solely suitable for soil analysis. Once again, the generated system is immovable by itself. Humans must transport it to the soil for testing. This system is not autonomous, and it is not associated with any robotic system. There have been numerous projects for ripe tomato detection, harvesting, and soil analysis. Most of the projects focus on either rover development or harvesting algorithms. However, in this project, we focus on a comprehensive system focusing on vegetable monitoring, ripeness detection using deep learning, automated harvesting, and growth monitoring using soil analysis.

There has been another paper that shows the importance of Convolution Neural Networks (CNNs) in addressing the fruit disease issue [8]. Recent advancements in CNNs show a higher accuracy as previous research mostly used handcrafted characteristics to classify fruit disease. Using a peach dataset, the paper presents a novel method that makes use of data augmentation, transfer learning, and MobileNetV2 architecture [8]. The CNN model performs well in the classification of Rot, Mildew, and Scab illnesses, with a Macro-average F1-score of 0.96 [8].

Here, this paper delves into the nexus between machine learning and machine vision for robotic grasping, with a focus on CNNs and their critical role in achieving high-precision target identification and recognition [9]. Large datasets provide challenges for effective traditional machine learning techniques, which is why unsupervised, self-supervised, and reinforcement learning have become more popular. The paper concludes that increasing robot grasp success rates in unstructured contexts requires the integration of vision and tactile feedback [9]. It offers an in-depth analysis of popular techniques, covering everything from conventional procedures to visual-tactile fusion, and offers insights into their development and constraints.

This paper [10] talks about enhancing farming efficiency by using robots as field operation assistants, following the tractor autonomously based on a leader-follower model. The system uses a combination of GPS and sensor-based methodologies for navigation and path following [10]. The experiments show the practical viability of the approach. On the other hand, these experiments need human supervision to conduct the operations. Also, this experiment will face challenges in real-time conditions like communication robustness, uneven terrain, etc. [10].

However, all the systems mentioned in the studies are developed to serve only one sole purpose (either detection algorithm or harvesting process). There have been no reports, to the best of our knowledge, on developing a robotic system capable of performing multiple tasks such as detect the ripe vegetables, harvest them, independently navigate around the field, and perform harvesting and soil analysis. The rover developed in this work can alleviate this limitation of previous relevant works. The use of the YOLOv5 algorithm instead of the more conventional two step detection algorithm allows our system to properly balance accuracy and speed. Also, we believe some upgradation will make the system useful for detecting disease and harvesting other kinds of vegetables or crops. The developed robotic system can be a great smart assistant to the farmers reducing manual pressure. The system will incorporate smart farming into Bangladeshi agriculture and help to improve the practicality and enjoyment of farming.

## Methodology

The basic goal of the project is to develop a robotics system that will intelligently harvest ripe vegetables using image processing techniques. The rover can be useful for harvesting different vegetables. For the testing and analysis purpose tomato has been chosen as the vegetable. The rover will have a mechanical chassis and a six-degree freedom-based arm. The arm will be used to do the harvesting and the soil analysis. The mathematical model known as "Inverse Kinematics" is used in this arm to make the end effector move to the desired location. For the soil analysis, there will be an NPK sensor connected to the arm that will give the soil nutrients value of Nitrogen, Phosphorus, and Potassium. Thus, the farmers can easily get to know the state of the soil health and take necessary measures accordingly. The rover can be used for controlled monitoring of the field. Like, as the camera used in the rover can give real-time data, this feature will give flexibility in using the rover for different sorts of navigation purposes. The rover can be controlled remotely via WIFI from any remote desktop as there will be a microprocessor on the control board. With the current state of cellular data availability in rural areas, we believe operating the system would not be disrupted in rural areas. However, the system is equipped with ports to connect on board modem and solar panel to ensure uninterrupted service for operating in remote areas. The microprocessor will relate to the microcontrollers which will operate the rover for operation purposes. As this rover is human controlled, this will have the adaptability to dynamic environments. Also, the non-autonomous behavior will result in less complexity in maintenance with lower cost while fulfilling the objectives. Farmers of a developing country like Bangladesh, equipped with a system having the features, would be able to significantly improve their production with less technical difficulties and moderate cost. We have divided the whole task into two segments, such as hardware development and deep learning-based image collection and analysis.

### C1. Hardware

For the electrical circuit connection development, different types of components have been used. There will be 2 different circuits connected with each other for 2 different purposes. One circuit will be for rover operation reasons, and the other will be used for soil analysis purposes. The detailed design of the circuit for these two operations is demonstrated below.

#### C.1.1 For a rover operation

**Fig 1:**
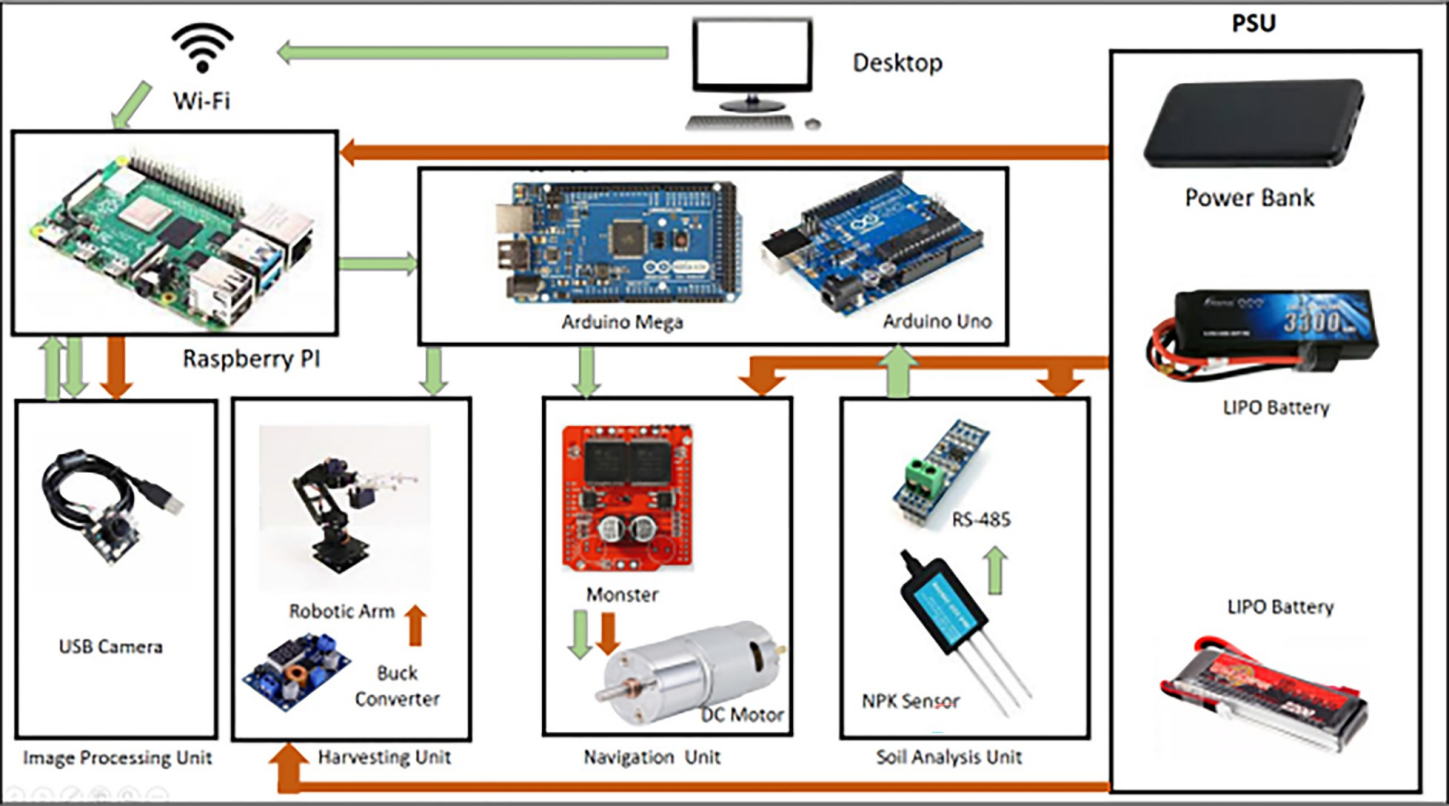
Component level diagram of the overall system.

In this rover system there are two motors. For the two motors, a monster motor driver has been used for driving the two motors. As the motor driver has two channels it can operate two motors at the same time. The motor driver will be connected to the controller. There will be six servos used for operating the arm. After analyzing it has been found out that, to operate the arm fully 6.5V power will be needed. So, there will be a 12V battery which will be connected to a buck converter to regulate the input voltage and will bring it down to 6.5V after converting. The motor driver and the buck converter will relate to the arduino microcontroller. Furthermore, the Arduino is connected to the Raspberry pi processor. Thus, the arm and the wheel of the rover have been connected for the rover operation purpose (Fig 1).

### C.2 Control system development

For the control system of the rover, onboard processors and controllers have been used which will control and will communicate with the user using a remote desktop via a wi-fi communication network. For the onboard processor, raspberry pi 4 is being used and for controlling purposes the Arduino Mega and UNO are being used.

**Fig 2:**
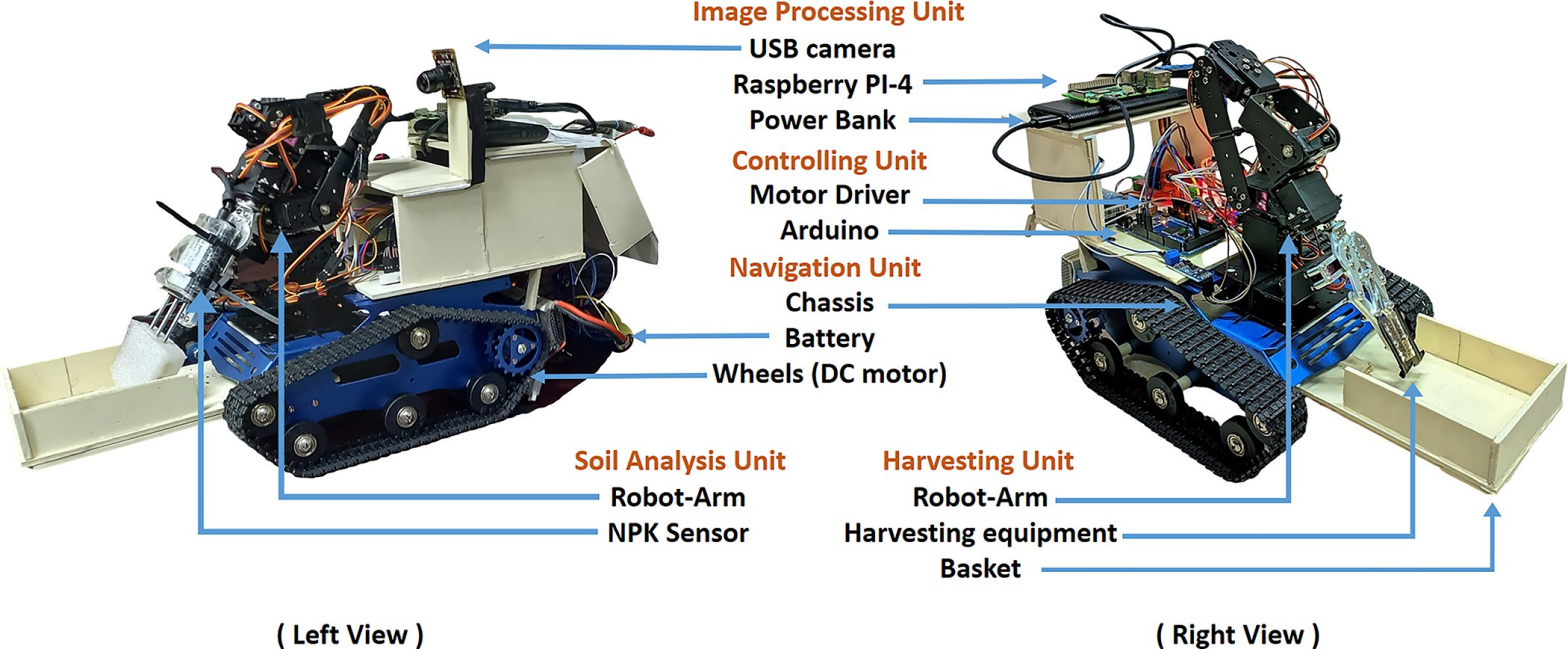
Developed rover bot including all units.

A clear overview of the functionality of the rover bot is demonstrated in Fig 2. How they are powered and how they are synergized. The Green arrows indicate the flow of signal and data while the red arrow indicates how each component is powered. The system consists of mainly four actuator sub-circuits. These include the image processing unit, harvesting unit, navigation unit & soil analysis unit. Again, the sub-circuit is controlled directly or indirectly by the microprocessor or through the micro-controllers. The microprocessor here (Raspberry PI) can be controlled remotely through a WIFI network. This makes the system wirelessly controllable. The system will also carry a power bank to keep the microprocessor running. Coming to the subsystems, the Image processing unit which consists of a USB camera is directly controlled by the microprocessor, unlike the other sub-circuit. In terms of navigation of our Rover Bot, the DC motor on the conveyor belt on both sides enables our system to move in a 2D plane. These motors are powered by the LIPO battery and signaled by the Arduino Mega microcontroller. However, they are driven by a Motor driver (Monster) instead of a microcontroller as they require more power than the microcontroller itself can provide. The NPK sensor transmits the soil data to the microcontroller in a soil analysis device. However, the soil data must first be transformed so that the microcontroller can understand it. This is done by the RS-485 module which works as an interpreter between the microcontroller and the NPK sensor. Again, like the Navigation unit, the Soil analysis unit is also powered by a LIPO battery. The Robotic arm which is the main component of the harvesting unit is controlled by Arduino Mega. The 6-servo motors connected to it are being controlled by the MCU. The figure shows the developed prototype of the rover bot (Fig 2).

### C.3 Rover arm movement using inverse kinematics

#### C.3.1 Robot arm specification

**Fig 3:**
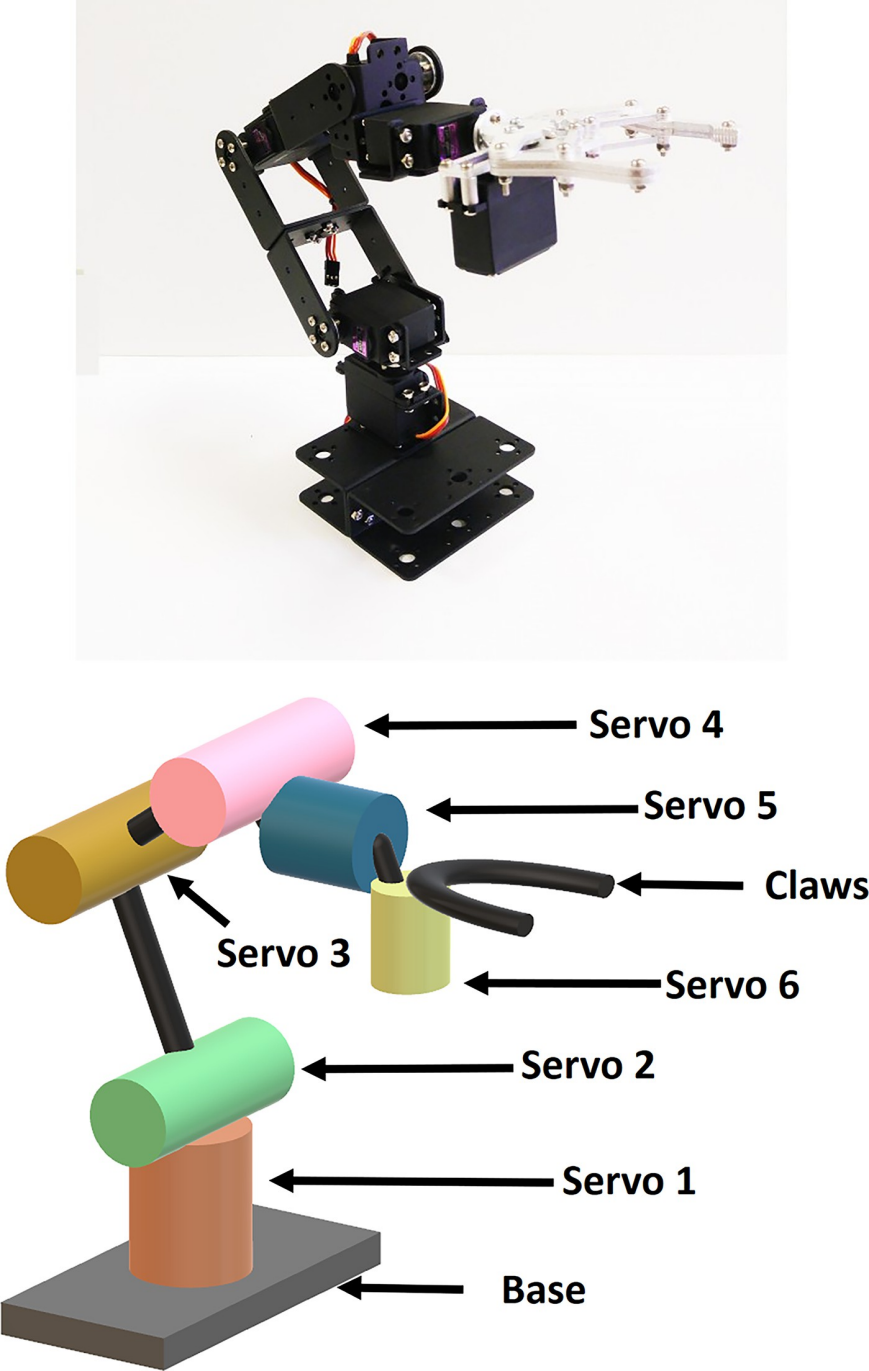
6 DOF robotic arm (a) prototype:(physical) image (b) 3D-diagram.

- Mechanical Arm: For the mechanical arm of the rover, a six DOF arm is being used. The arm is made of aluminum. To operate the mechanical arm there will be 6 servos connected with every degree of freedom. There is a 2-finger claw attached to the arm for harvesting purposes, as shown in Fig 3. The claw has 2 blades attached to the fingers so that it can cut the branch easily. The arm joint and motor specifications are shown in Tables 1 and 2.
- Arm Motor: For the arm motor MG996R servo has been used.

**Table 1:** Joint specifications.

Servo Number	Link Length (*a*_*i*_ (cm))	Link Twist (*α*_*i*_ (Deg))	Link Offset (*d*_*i*_ (cm))	Joint Angle (*ϴ*_*i*_ (Deg))
1	0	-90	9	-120< *ϴ*_*0*_ <120
2	10.5	0	0	-30< *ϴ*_*1*_ <120
3	10	0	0	-160< *ϴ*_*2*_ <160
4	5.5	90	2.5	-160< *ϴ*_*3*_ <160
5	3	-90	0	-180< *ϴ*_*4*_ <180
6	8	0	0	0< *ϴ*_*5*_ <35

**Table 2:** Robot arm motor specifications.

Specifications	Values
Dimension	40.7×19.7×42.9 mm
Stall torque	9.4 kg/cm (4.8V); 11 kg/cm (6.0V)
Operation speed	0.19 sec/60 degree (4.8V); 0.15 sec/60 degree (6.0V)
Operating voltage	4.8 ~ 6.6V
Gear Type	Metal gear
Dead band width	1μs
Wire length	32cm

**Moving End-Effector to desired position:** To make the Robotic-arm end-effector (claw that plucks the tomatoes) reach its desired position we are required to apply Kinematic.

Here is our case as our input is the Cartesian coordinates of the detected tomatoes and we need our Robotic-arm’s claw reach the tomatoes by orienting the servo motor positions, hence we need to implement a proper inverse kinematics model.

**Fig 4:**
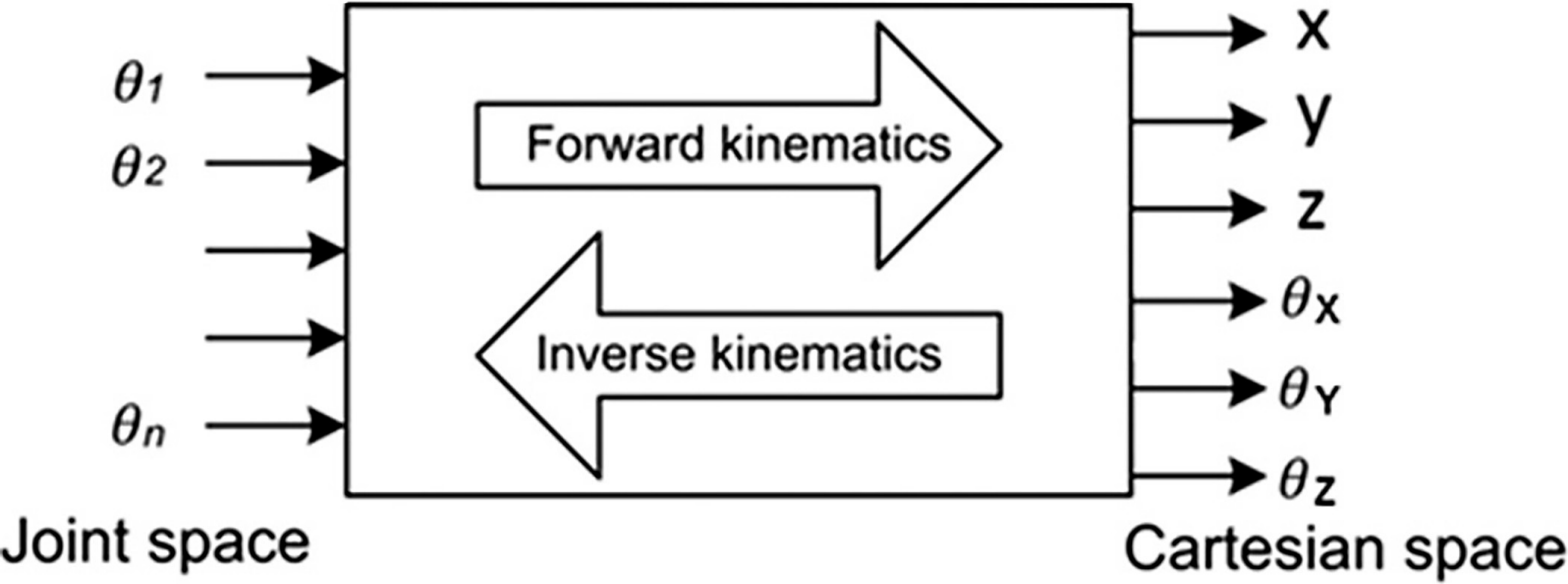
Kinematics types based on Joint & Cartesian space variable transformation [11].

Inverse Kinematics: Before we applied inverse kinematic technique to our robotic arm, we considered possible Inverse Kinematic Techniques used traditionally. The basics of Joint based and Cartesian space transformation methods are illustrated in Fig 4.

To solve joint-space variables, the inverse-kinematics solution technique is mainly of two types, analytical and numerical solution [12].

The analytical method is basically a closed-form solution method, which yields the joint-space variable value given cartesian values using mathematical equations. Hence, this type of Inverse-Kinematics technique is computationally less expensive.

In contrast, numerical methods are computationally more expensive as these are iterative approaches not yielding any exact solution. However, the numerical method is useful when the analytical method is either too complex or it is impossible to find an exact solution for a particular manipulator model. There are two types of numerical methods, including Newton-Rahpson method and different gradient based nonlinear algorithms [12]. Moreover, other iterative methods also include neural networks [13,14], evolutionary computing [15,16] etc.

Here, for our robot-arm manipulator we used analytical methods to solve inverse kinematics instead of going for numerical methods. The reason behind this is that numerical methods of inverse kinematics techniques have a huge computational overhead due to numerical calculation of gradient vectors and high nonlinearity [12]. On the other hand, for a high degree of freedom (DOF) of the manipulator, an analytic solution is unsolvable or very difficult to find [17]. Even if a solution exists it may not yield a single valued output as in most cases it is a multi-valued function [18].

Path planning and navigation: In this section we will explore how we deduced the inverse-kinematics model to guide the Robot-arm’s end effector to reach the given cartesian-coordinate.

Since the inverse-kinematics model we are using relies on an analytical based closed-form solution, hence it is important to note that to deduce a single valued inverse-kinematics solution, we were to specify a few initial conditions. The analytical solution of the inverse kinematics equation for such a high degree of freedom would offer infinitely many possible solutions. This term is known as “Kinematics redundancy”. Hence, this would introduce the need for further optimization to generate optimal manipulator configurations in a particular scenario.

However, we opted to reduce the computational complexity for the 6-dof robot arm and curtail the possible solution into a single valued solution that would be a satisfactory sub-optimal solution in all the use cases we aim for. Hence, here we demonstrate how we can abate the workload of the microprocessor by reducing the computational overhead it had to go through for an optimization-based approach of inverse kinematics otherwise.

Firstly, let’s denote the six servos as S_1_, S_2_,..,S_6_ and their corresponding angles as *θ*_*1*_, *θ*_*2*_….,*θ*_*6*_.Now S_5_, S_6_ is used to rotate or open-close the claw attached to the robot arm. Hence, having no impact on end effectors position for different values of *θ*_*5*_, *θ*_*6*_. Therefore, this portion is influenced by the angle *θ*_*4*_ of servo S_4_. To summarize, we have three space variables x,y,z and four joint space variables *θ*_*1*_,*θ*_*2*_,*θ*_*3*_,*θ*_*4*_ to be accounted for in our inverse kinematics model. As a result, to establish a proper inverse-kinematics model for the robot-arm, we would require equations for *θ*_*1*_-*θ*_*4*_ values from cartesian coordinate values. Moreover, to avoid getting infinite solutions, we impose an initial condition such that *θ*_*4*_ remains parallel to ground (base).

**Fig 5:**
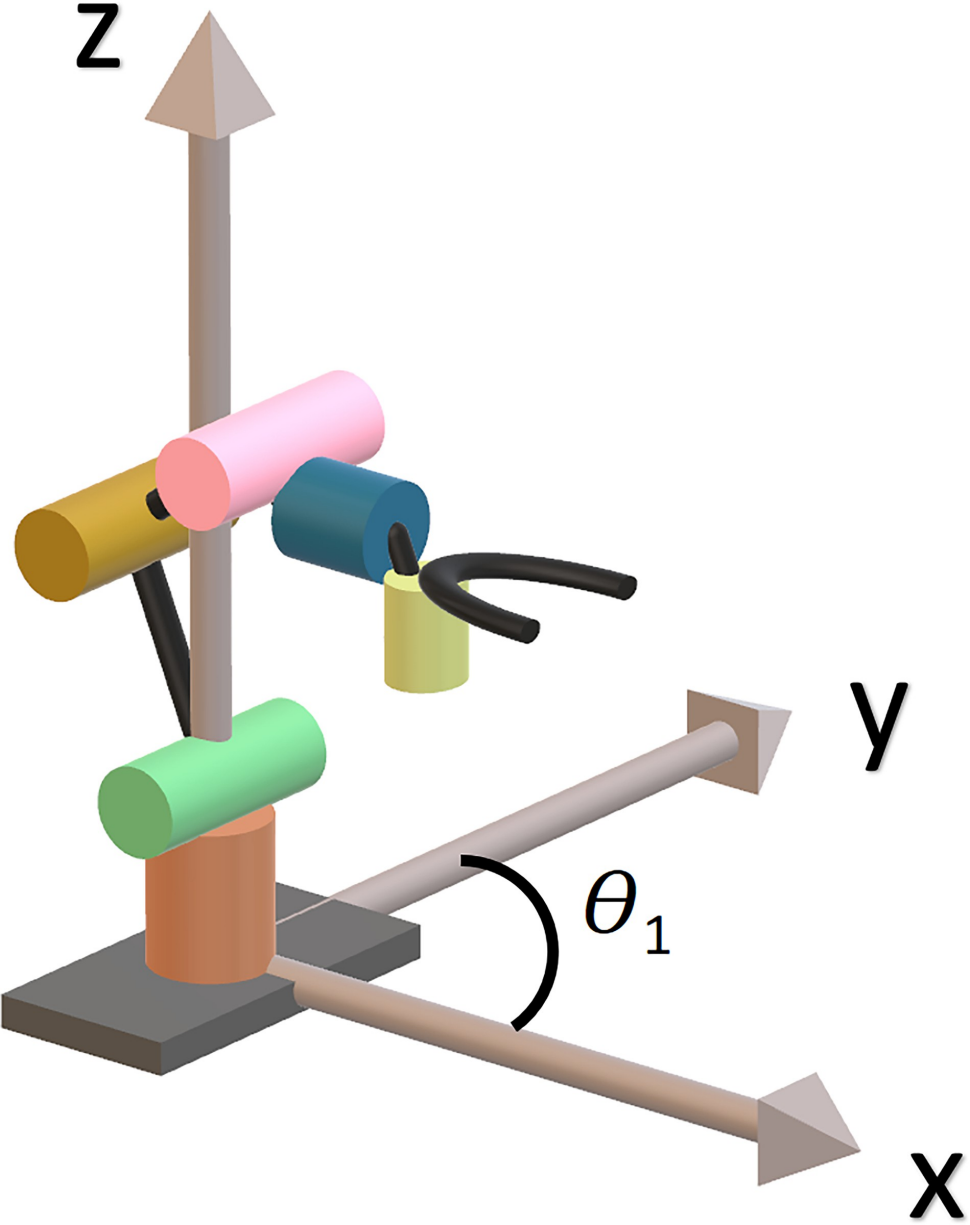
Horizontal plane movement illustration (Cartesian axes along the manipulator).


Among the four angle values of joint space variable, *θ*_*1*_ can be easily found using cartesian x,y values as it controls the rotation of the whole arm in x,y plane (Fig 5).

$$Ɵ=tan^{‐1}(\frac{y}{x})$$ (1)

**Fig 6:**
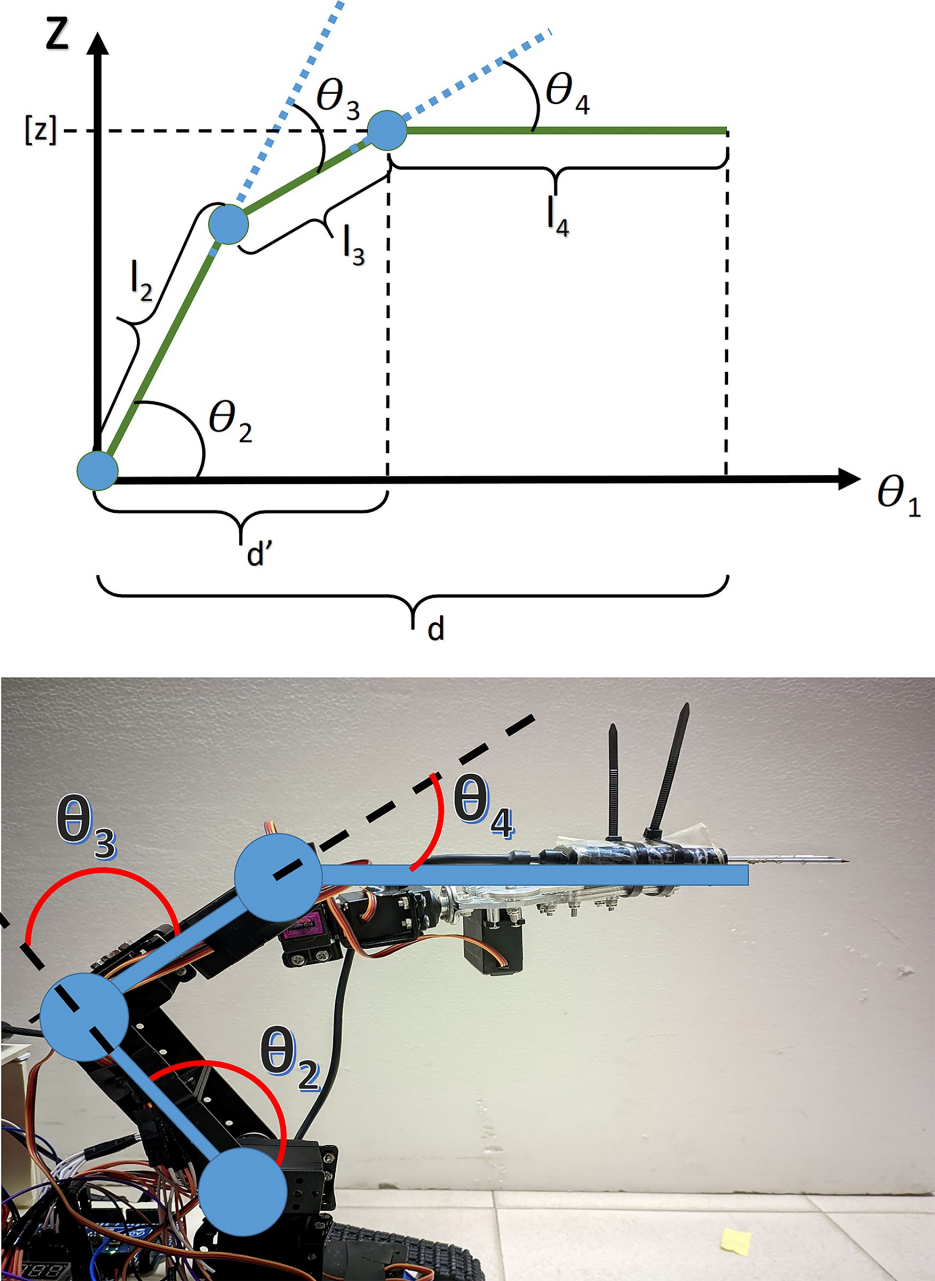
Vertical plane movement illustration.


As, *θ*_*1*_ rotates the entire vertical plane servos of the robot-arm, let the new base length of the vertical plane is d. (Fig 6A).

$$d=\sqrt{x^{2}+y^{2}}$$ (2)

In order to determine *θ*_*2*_, *θ*_*3*_ and *θ*_*4*_ we initialize an initial condition that *θ*_*4*_ remains parallel to ground (so that claw remain in an appropriate position to pluck tomatoes) (Fig 6A).

Hence,

$${ϴ}_{4}=-{ϴ}_{2}-{ϴ}_{3}$$ (3)

To solve for the final two angles θ2 and θ3. let

$$d\prime =d-l_{4}$$ (4)

Now using trigonometric equation, we can solve for *θ*_*2*_ and *θ*_*3*_ (Fig 6A)

$${ϴ}_{3}=-\pi +(cos^{-1}((-{d\prime }^{2}-z^{2}+l_{2}^{2}+l_{3}^{2})/(2l_{2}l_{3})))$$ (5)

$${ϴ}_{2}={tan}^{-1}(z/d\prime )-{tan}^{-1}((l_{3}\;sin\;({ϴ}_{3}))/(l_{2}+l_{3}\;cos\;({ϴ}_{3})))$$ (6)

Hence, for given x,y,z coordinates, solving for *θ*_*1*_, *θ*_*2*_, *θ*_*3*_ and *θ*_*4*_ would be sufficient to make the end-effector (the claw) of the Robot-arm reach the desired position.

## Deep learning (Yolov5) based image analysis

In this project, one of the requirements is detecting the ripe vegetable for harvesting purposes. For detecting the ripe vegetable, an image processing method will be used. In image processing, a model will be trained to detect the expected object. The steps of performing image processing for ripe tomato detection are given below.

### D.1 Creating dataset

**Fig 7:**
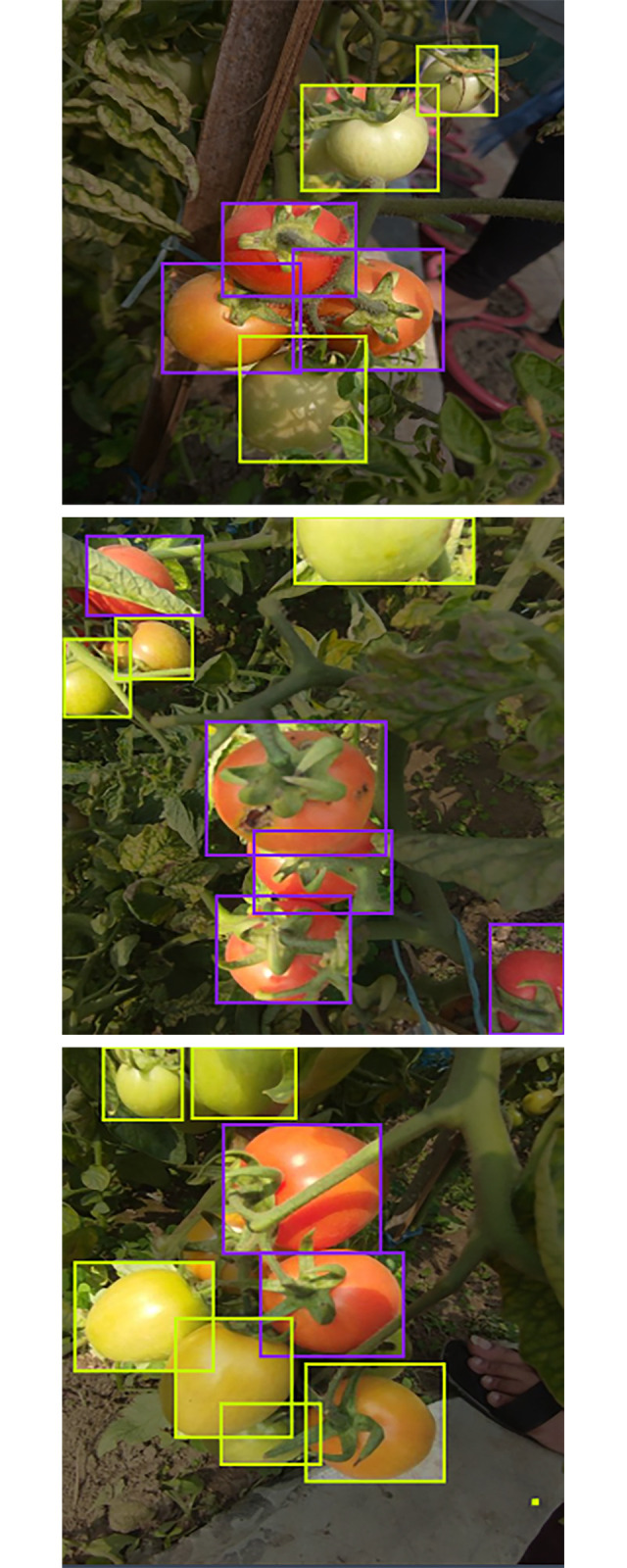
(a) Image for training set, (b) image for validation, (c) image for testing.

First, a dataset has been prepared for training the system containing 500 images. The images were taken from a tomato field. The images were divided into three parts. Among the 500 images there are 350 images as training set, 100 images as validation set and 50 images as testing set. Here the ratio for the training set, validation set, and testing set is 70%, 20% and 10% accordingly. The images below show the dataset images for training set, validation set and testing set (Fig 7).

### D.2 Labeling and annotation

After selecting the dataset the images need to be labeled and annotated for further training purposes. In this project, the ripe tomatoes have been annotated as ‘0’ and the unripe tomatoes have been annotated as ‘1’.

### D.3 Selecting a model

**Fig 8:**
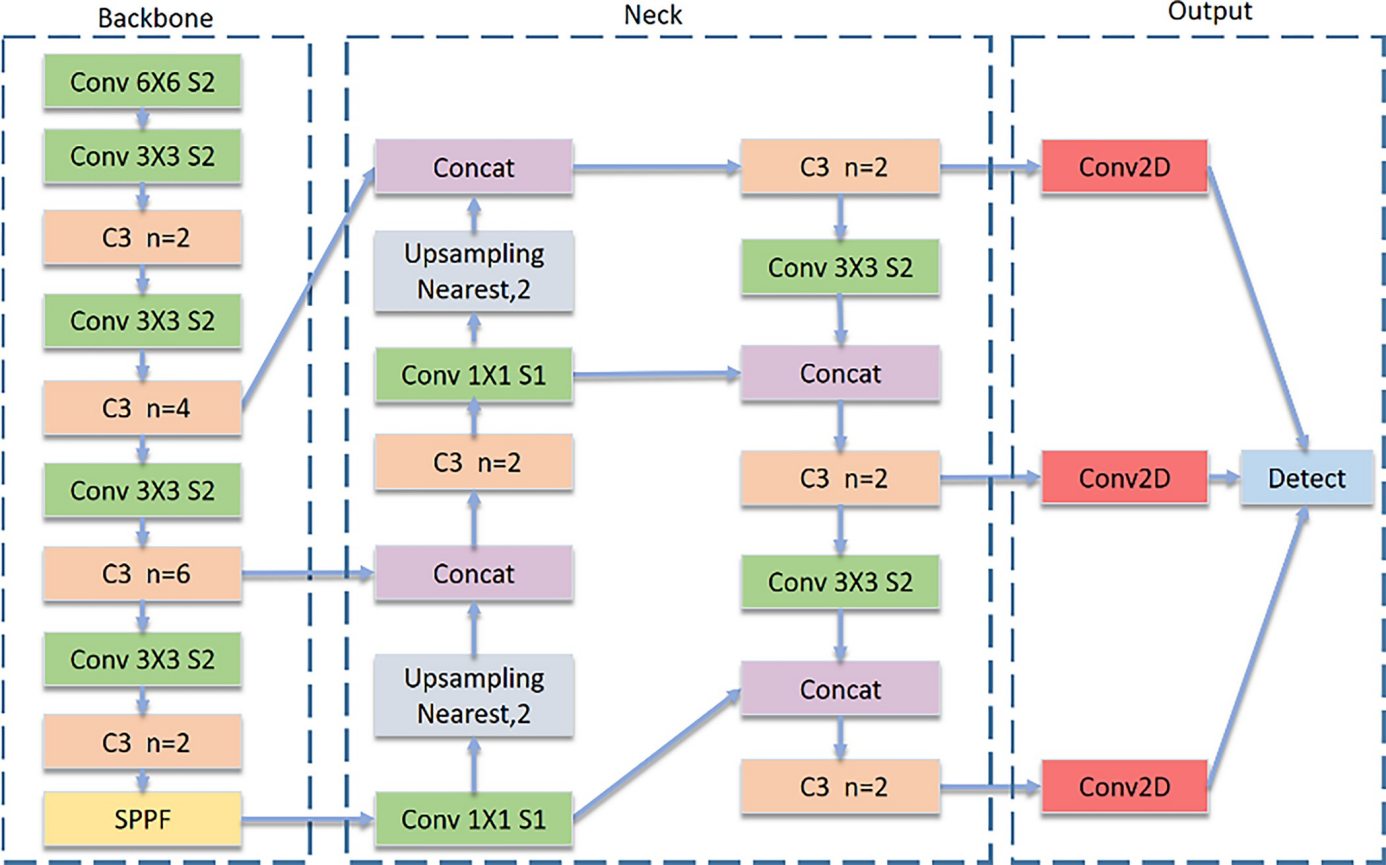
YOLO V5 model architecture [21].

There are two types of object detection techniques: single-stage detectors and two-stage detectors. The first step of a two-stage detector is to use a selective search to extract the candidate regions containing the target objects from the input image. The second step is to use regression analysis to classify the extracted candidate region [19]. Usually, the two-stage detectors consist of an R-CNN model series. In contrast, the single stage detector obtains both localization and object categorization (classification) information in one step, skipping the candidate region extraction phase. SSD, YOLO, and Detect Net are the single-stage object detectors. The paper suggests a method based on the YOLO object detection model as single-stage object detectors perform better in inference than two-stage detectors [19]. In this project, YOLOv5n has been used as the training algorithm. Compared to the other algorithm, this one has better performance in object detection. As we are going to do real time object detection, YOLO can do it in a single pass through the neural network, that is why this is incredibly fast and efficient as compared to the two-stage detectors [19]. This method significantly alleviates the computational workload and delivers substantial enhancements in speed. Moreover, among YOLO models YOLOv5 is the most efficient model compared to its previous versions with better precision and speed, reduced size and being implemented in an open-source machine learning framework named PyTorch [20]. However, the latest version of YOLO includes YOLOv6 and YOLOv7. However, they were developed by independent developers around the world in June and July 2022, hence YOLOv5 is the latest officially released version of the YOLO model [20]. Furthermore, YOLOv5 greatly improves applications like trained neural networks and real time video processing by enabling it to run on weaker devices [20]. Which perfectly aligns with our goals (Fig 8).

### D.4 Training

**Fig 9:**
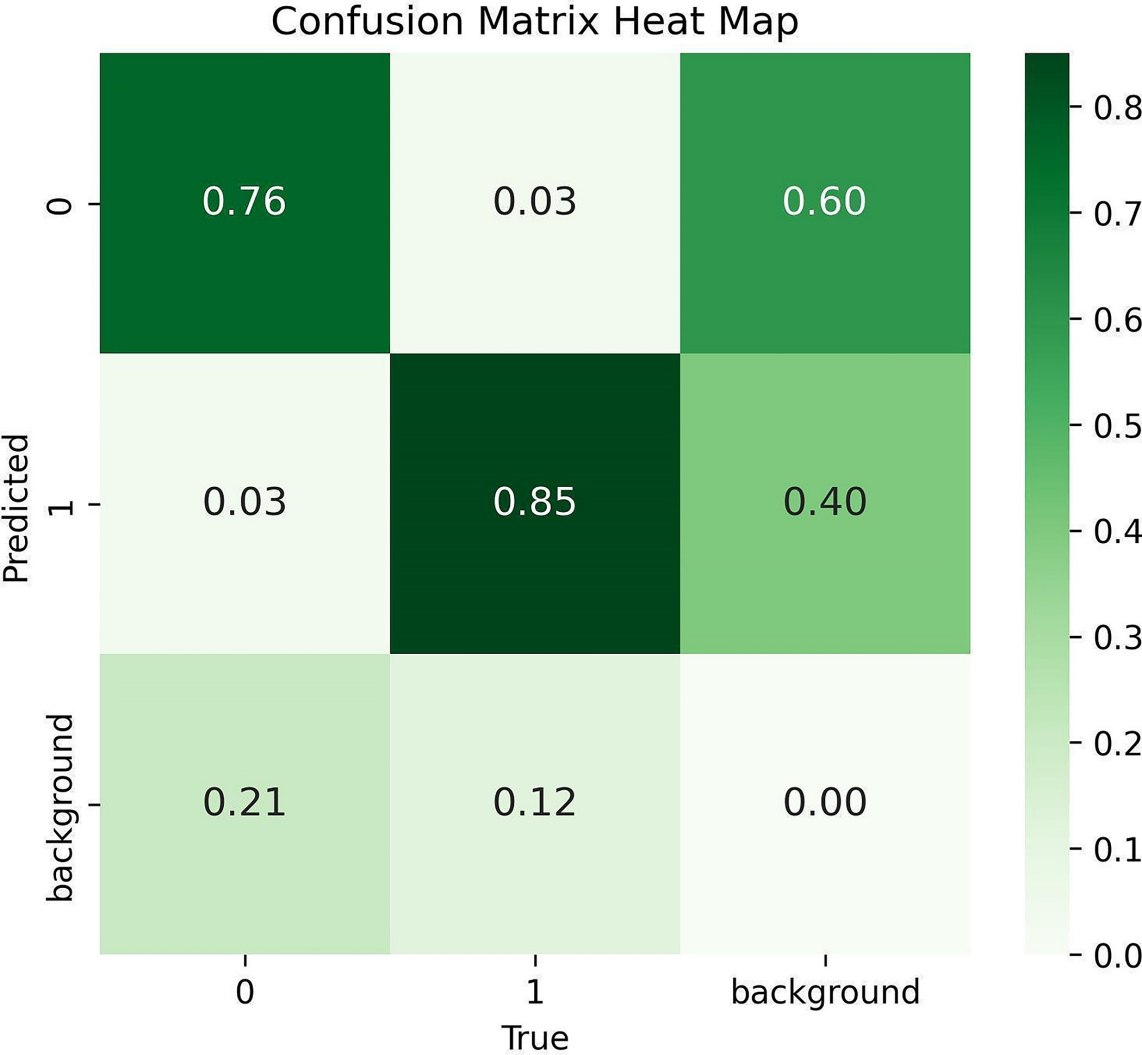
Confusion matrix.

**Fig 10:**
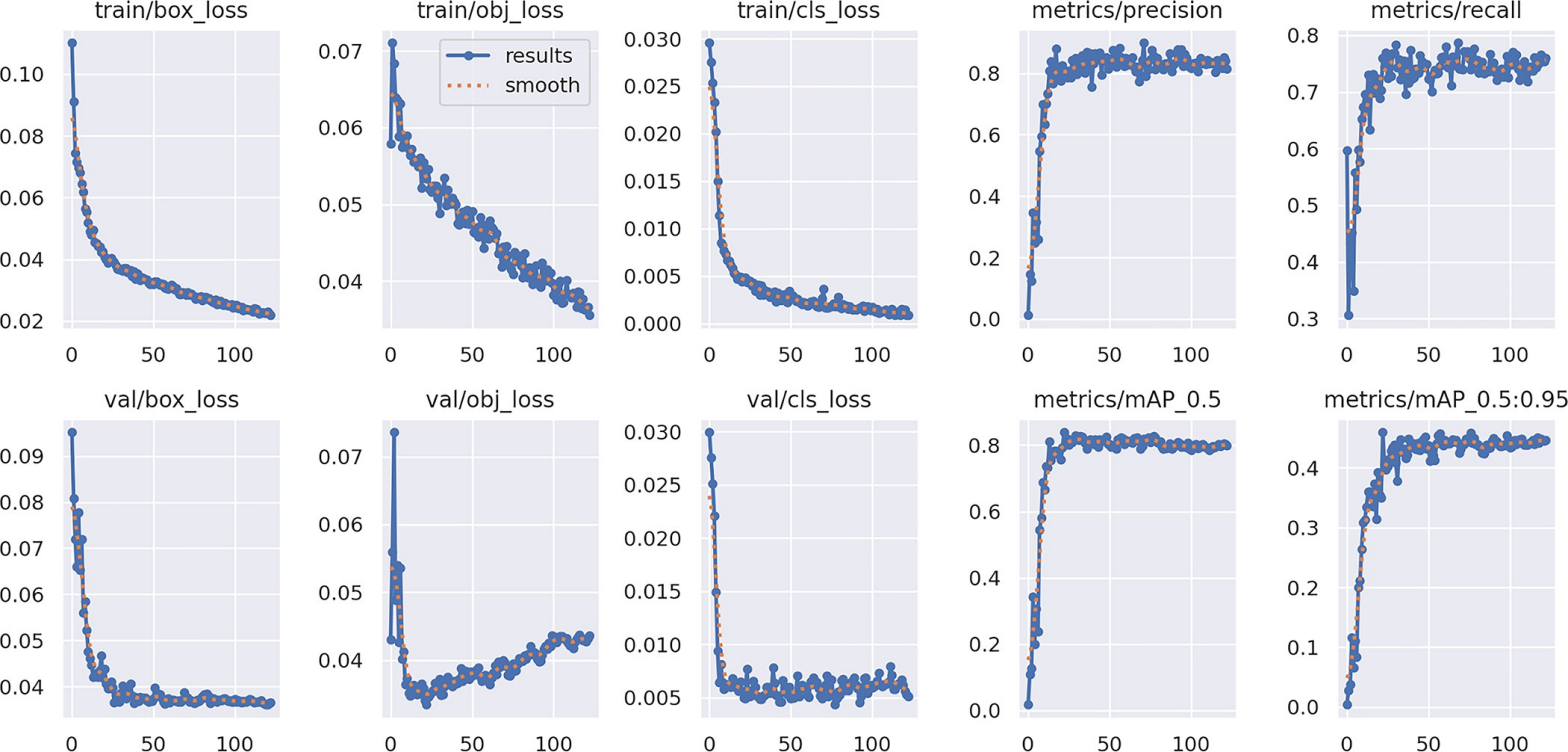
Plots of the training epochs for the training and validation set for box loss, objectless loss, classification loss, precision, recall, and mean average precision (mAP).

For training this model Google Colab has been used that provides free access to powerful GPUs and requires no configuration. To train the model, 150 epochs have been used, which tested the training performances and increased the rate of precision. The confusion matrices of the model have been shown (Fig 9). The improvement in the model has been shown in the graphs in Fig 10 which display the precision and loss values.

The figure above displays three basic forms of loss: classification loss, objectless loss, and box loss (Fig 10). The box loss measures how accurately the algorithm can locate an object’s center and how completely the predicted bounding box covers an object. The chance that an object exists in a proposed region of interest is basically measured by object lessness. If the objectivity is high, an item is probably present in the image window. Classification loss indicates how effectively the algorithm can predict the proper class of an object [22]. After the evaluation, the model provides a precision score of 0.8518, recall score of 0.7624. As well as the mean average precision (mAP) scores of 0.8213 and 0.4419 for @0.5IoU (Intersection over Union) and @0.95IoU respectively. The definition of these performance parameters can be found in Section 1 of the Supplemental Document. This result validates the approach’s ability to predict ripe tomatoes clustered in a variety of situations properly. The confusion matrix is shown in Fig 9. Here, the true positive value is 0.85 and the true negative value is 0.03, while the false positive value is 0.76 and the false negative value is 0.03. The model rapidly enhanced its precision, recall, and mean average precision metrics, displaying significant improvement until approximately 150 epochs. During this period, the losses associated with bounding box, objectless, and classification for the validation data exhibited a sharp decline. Beyond around epoch 150, the model’s performance plateaued. Also, there is an indication of the model overfitting the training data, quantified by an increase in the difference between training and validation loss around 150 epochs. Early stopping was implemented to identify and retain the most optimal weights during training. We have also calculated the F1-confidence and precision-recall characteristics of the trained model in S1 and S2 Figs in S1 File. The related equation, and confusion matrices are given in S1 File.

### D.5 Deployment and Evaluation

**Fig 11:**
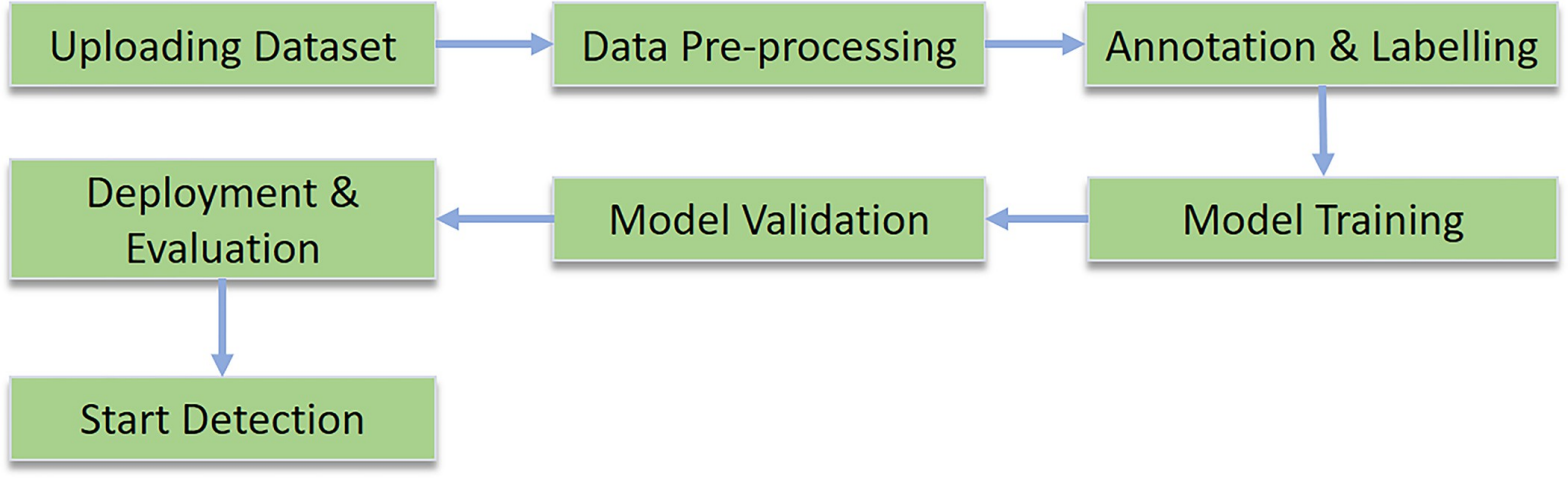
Workflow diagram of image processing.

After training the model, the weight file will be used to deploy the model in any processor for further evaluation. After deploying the system can-do real-time analysis in any processor. As the Raspberry Pi 4 is being used here as the processor the necessary environment has been created with all necessary software to operate the onboard image processing. The basic block diagram of the on-board image processing system is shown in Fig 11. After that, the system will be fully functional for object detection.

## Performance analysis

### E.1 Detecting ripe vegetables

Ripeness detection is one of our main objectives. After doing the ripeness detection the system will proceed to the harvesting process. We can do the evaluation of the model that has been used for training the model. For evaluation purposes, the precision curve and the loss curve we obtained can be used (Fig 10). After completing the training, we got the precision curve and the loss curve. These two curves describe how well the system is performing while detecting the ripe vegetables.

After deploying the model in the processor when we use the USB camera, we get the feed for object detection something like this. For the evaluation purpose we created an environment where we put some ripe and unripe tomatoes together so that we can verify if the system works properly when many tomatoes are in a bunch.

**Fig 12:**
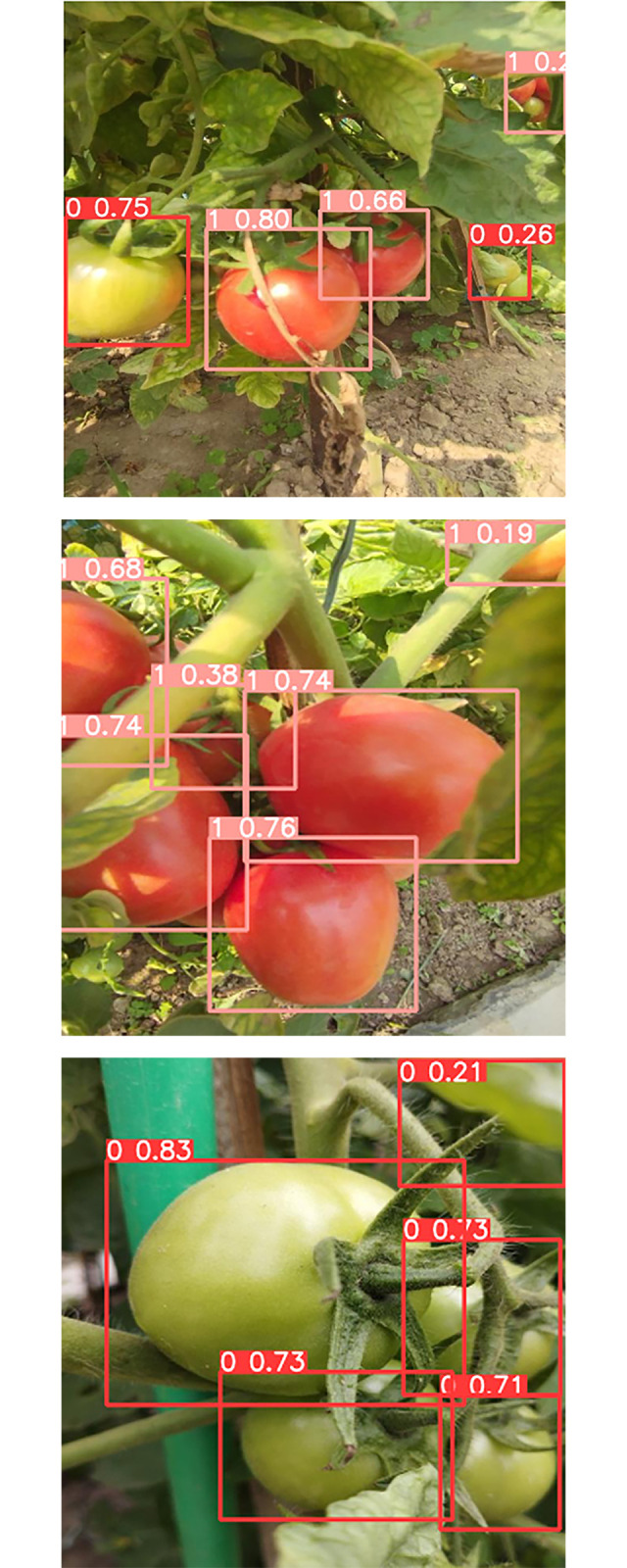
Ripe (0) and unripe (1) tomato detection based on IOU threshold values (0 to 1).

Whenever the system sees ripe tomatoes, it will label it as ‘0’ and whenever it will see an unripe tomato it will label it as ‘1’. The system is also skilled at detecting the color intensity of the object. The parameter that is shown along with the labeling parameter is the color intensity value. In this figure, we can see the color intensity value of the very ripe tomatoes is more than 0.7(Fig 12). The same goes for the unripe tomatoes as well. By checking the color intensity value, we can easily determine which tomatoes are ready to harvest. Furthermore, since our objective is to identify ripe tomatoes, the similarity in color between unripe tomatoes and leaves will not pose a challenge. Additionally, given that the dataset comprises images captured in real field conditions, the model has been trained to account for situations where the color of leaves and sunlight does not interfere with the accurate detection of ripe tomatoes.

### E.2 Harvesting

**Fig 13:**
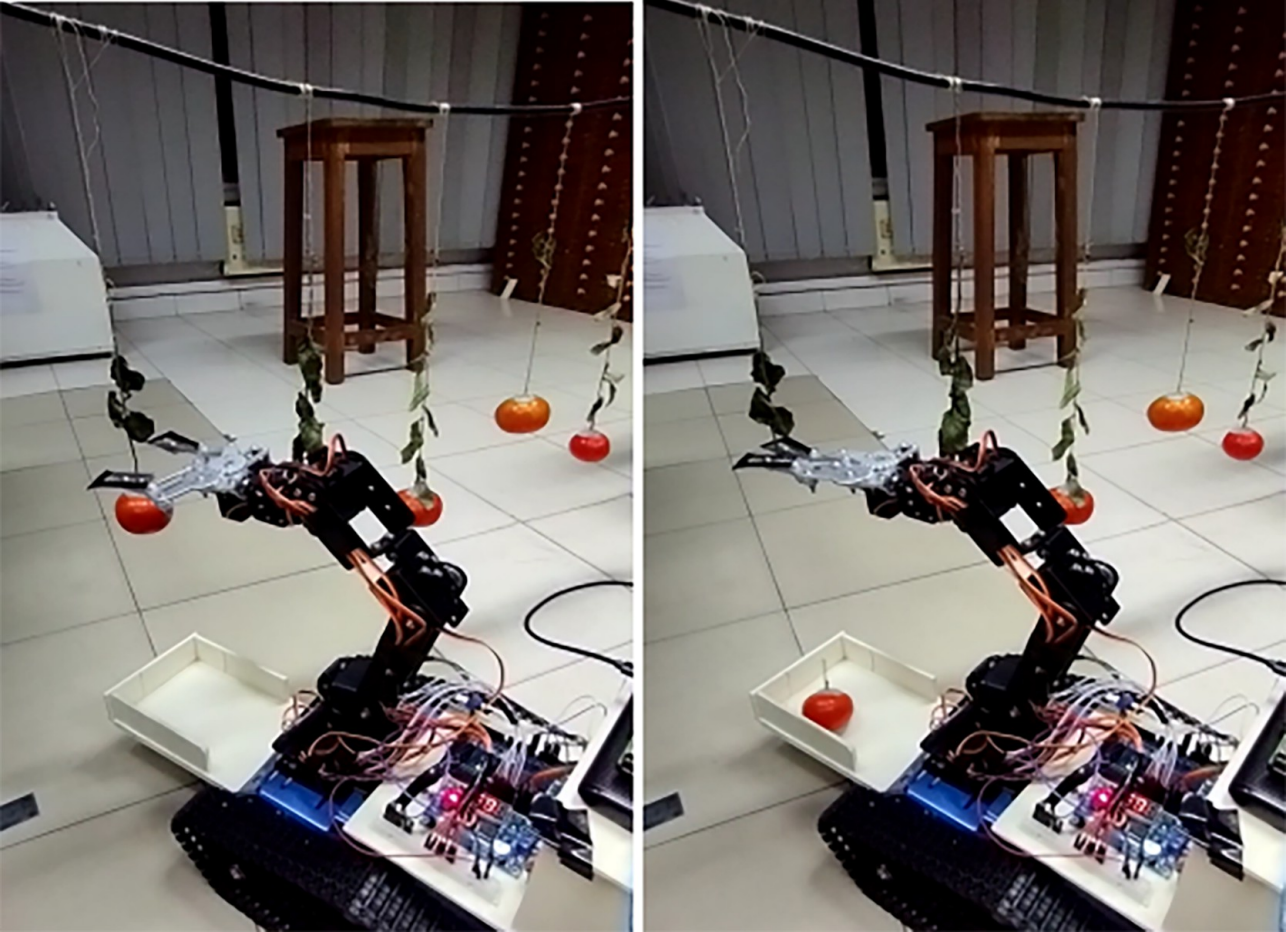
Harvesting process (a) before harvesting ripe tomato, (b) after harvesting ripe tomato.

For evaluating the harvesting procedure, we created an environment in a room where we strung together some ripe and unripe tomatoes using some brunches. To identify the tomatoes, the rover first went through the room. Once it had located the tomatoes, it differentiated ripe and unripe tomatoes. Upon detecting a ripe tomato, it moved closer to that tomato and started harvesting. That was the adjustment we made to assess the rover’s harvesting procedure. Now, elaborating on the whole harvesting process, first the rover will detect the ripe tomato. Upon detection of the ripe tomato, the Roverbot will harvest the ripe tomatoes. The actuator for this process would be the 6 degrees of freedom-based robot arm inserted over the Rover-body. The process is illustrated in Fig 13.

The robotic arm is equipped with claws to pluck the ripe tomatoes. The claw here is a two-finger claw using the ‘v’ mechanism. Here, blades relate to the claws for cutting the branches of the ripe tomatoes. There will be a servo motor connected to the claws which will help to open and close the claws. By operating this servo motor the branches of the tomatoes will be cut and thus will be plucked. There will be a basket connected to the rover body which will collect the plucked tomato. Since this is a prototype of the project, the current attachment of the basket is designed in this manner. However, for the final design, the basket will be significantly larger, and the claw will be responsible for harvesting the tomato and subsequently placing it into the basket. We may consider claws as an end effector for robotic arms. The rest of the servo motor will help the arm reach its destination. Now the problem arises when we try to make the robotic arm’s end effector reach a certain point. We are required to translate the coordinate value to joint space or angle values of the servo motor, to make the 6-servo motors reach the destination point altogether. To ensure this we are required to apply a mathematical concept called ‘Inverse kinematics to make the end effector of the Robotic arm reach the position. This requires all the servo motors to synchronize properly.

### E.3 Soil analysis

Testing soil samples to ascertain their physical and chemical characteristics is the process of soil analysis. Soil analysis is a crucial component of soil science that is used to evaluate the soil’s fertility. For our system, we will be only measuring the vital soil nutrients which are Nitrogen (N), Phosphorus (P), and Potassium (K) using an NPK probe. The electrical conductivity of the soil, which is impacted by the presence of these elements, is measured using the NPK probe. A reading is taken once the probe has been put into the ground. The sensor probes are sufficiently sharp to conduct tests in all soil types. The sensor can reach a depth equivalent to the length of its probes within the soil, and the arm is equipped to apply adequate force for this purpose. Multiple tests have been conducted to validate this assertion. To assess the quantities of nitrogen and phosphorus, the reading is then compared to a chart [23, Table 2] which is shown below.

Here, in Table 3, the values of NPK are given in Kg/ha but from the NPK sensor we get the values of soil in mg/Kg unit. Given this, we can calculate the NPK ratio to determine the soil’s quality. Additionally, the amount of nutrients in the soil differs from one soil to another. Furthermore, it depends on the state of cultivation. Crop yield and general soil health are improved by a 4:2:1 NPK ratio in the soil [24].

**Table 3:** Rating chart for soil test data [21].

Nutrient	Low	Medium	High
Available nitrogen (N)	< 240 Kg/ha	240–480 kg/ha	> 480 Kg/ha
Available phosphorus (P)	< 11.0 Kg/ha	11–22 Kg/ha	> 22 Kg/ha
Available potassium (K)	< 110 Kg/ha	110–280 Kg/ha	> 280 Kg/ha

We ensured the task’s functionality by constructing a specific environment where the NPK data was taken from a flowerpot soil and carried out the soil analysis there.

The NPK sensor basically operates at 12 volts. So, we used a 12V, 3300mAh LiPo battery to power the sensor. Using the 6DOF-based arm, the probes of the sensor are dipped in the soil, and it collects the data. But as the data is in analog form, the RS485 module converts it to digital data so that Arduino UNO can process it.

**Fig 14:**
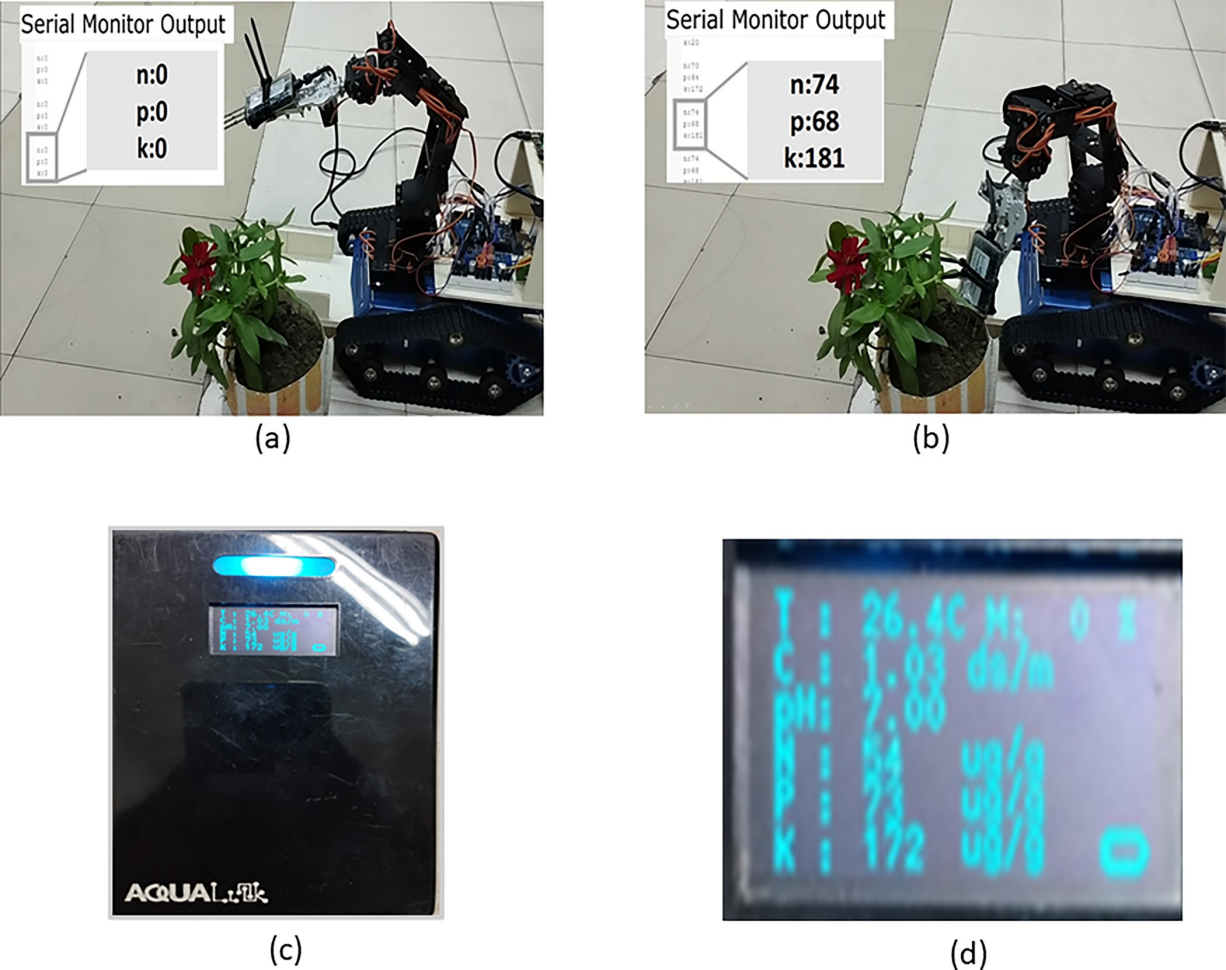
(a) NPK sensor and Serial Monitor (Before dipping in the soil, (b) NPK sensor and Serial Monitor (After dipping in the soil) (c) Aqualink soil analysis data and (d) Aqualink soil analysis data close-up.

From Fig 14 (A) we can see that the probe has not plunged into soil yet. That is why in the serial monitor of a remote desktop, we can see that the values of nitrogen, phosphorus, and potassium are zero.

From Fig 14(B) we can see that the probe is plunged in soil, and in the serial monitor of our remote desktop, the values of Nitrogen (N), Phosphorus (P), and Potassium (K). The values of soil nutrients are given below.

From Fig 14(C), we can see nitrogen, phosphorus, and potassium values from the same soil. The values of soil nutrients are given below (Fig 14).

Table 4 indicates the recorded nitrogen, phosphorus and potassium value of tested soil using our NPK probe and with commercially available probe (Aqua link). The comparison table indicates our probe can efficiently identify the status of the soil (low, medium and high) similar to the commercial probe confirming the efficacy of the device.

**Table 4:** Collected data from NPK sensor and marketed NPK device.

Soil components	Data from NPK sensor (mg/kg)	Data from aqualink NPK device (mg/kg)
Nitrogen	74	54
Phosphorus	68	73
Potassium	181	172

NPK sensors provide values in a comparable range, as can be seen by comparing the two results. The NPK probes’ depth in the soil is the main cause of the values’ variance. However, by utilizing this technique, we can estimate the quantity of nutrients in the soil.

## Challenges and future work

### F. 1 Challenges

Robustness in Unstructured Environments: Robustness in Unstructured Environments: Farming fields are intricate, unstructured environments with a diversity of crops, unforeseen barriers, and shifting lighting conditions. Agricultural robots must be robust and adaptable to handle these shifting conditions and guarantee accurate and efficient harvesting, growth monitoring, and soil analysis. The suggested rover is capable of navigating through unstructured rocky terrain, utilizing a track system up to a certain level. Nevertheless, challenges may arise when the system operates in muddy or wet soil conditions.

Crop Detection and Classification: Although YOLOv5 has shown good results in object detection, it can still be challenging to accurately identify and classify different crop types, especially when crops are at different growth stages or are hidden by vegetation. If you wish to improve the model’s performance, you must train it on a variety of representative datasets.

Real-Time Processing: For autonomous harvesting, growth monitoring, and soil analysis, real-time processing abilities are required. The YOLOv5 implementation can be computationally demanding and may require modification to achieve real-time performance on robots with constrained resources.

Data Privacy and Security: Agriculture-related data are crucial and delicate, such as growth rates and the results of soil analyses. To gain farmers’ trust and protect their sensitive data, using YOLOv5 and cloud-based analysis tools needs maintaining data privacy and security.

Energy Efficiency: Robots used in agriculture usually operate in remote areas with little access to power sources. It is crucial to ensure energy-efficient operation over extended periods of time to reduce the need for frequent recharging or refilling.

Human-Automation Interaction: Safety, coordination, and communication issues must be fixed to combine agricultural robots with human laborers. The relationship between humans and robots must be seamless to create profitable and effective farming techniques.

### F.2 Future work

Enhanced Object Detection Models: Object recognition models like YOLOv5 may be continuously enhanced through research and development to perform better at accurately identifying and classifying various crop varieties. This involves examining cutting-edge designs and making use of larger and more varied datasets to address specific issues in agriculture.

Multi-Modal Sensing: Combining data from several sensing modalities, such as RGB, multispectral, and LiDAR data, allows for a more comprehensive picture of crop health and soil properties. Combining these data sources with YOLOv5 allows you to make agricultural decisions with more understanding.

Edge Computing and On-Device AI Investigating edge computing and AI capabilities on-device can assist reduce reliance on cloud-based processing for real-time applications. YOLOv5 edge deployment optimization can improve agricultural robot autonomy and reaction.

Autonomous Navigation and Path Planning: Advances in autonomous navigation and path planning algorithms may allow agricultural robots to traverse fields more effectively, avoiding obstacles and improving harvesting routes.

Integration of AI and Robotics: Deeper integration of AI algorithms with robotic hardware could lead to more complex and flexible agricultural robots. This includes creating harvest-specific robotic arms and grippers, as well as sensor payloads for accurate growth monitoring and soil analysis.

Data Analytics and Decision Support: Modern data analytics and decision support systems that can analyze and interpret data from several sources can provide farmers with actionable insights, allowing them to make informed decisions to improve crop yields and soil health.

Finally, fixing the concerns and focusing on future work in agricultural robots and YOLOv5 can significantly boost the efficiency and efficacy of agricultural autonomous growth monitoring, soil analysis, and harvesting. The evolution of these technologies will be essential in shaping the future of precision agriculture and sustainable agricultural approaches.

## Conclusion

To conclude, the proposed study demonstrates the successful integration of deep learning techniques into an Agricultural Rover for vegetable harvesting and soil analysis. The rover integrates computer vision, robotics, and soil sensing technologies to perform autonomous tasks in agricultural fields. We conducted extensive field trials to assess the efficiency and accuracy of the rover in vegetable detection and harvesting, as well as its effectiveness in soil analysis. Despite numerous studies conducted on agricultural harvesting robots, this research proposes a robot that not only performs harvesting but also conducts soil analysis and serves for field monitoring, incorporating a camera for ripe tomato detection. This camera can also double as a security camera for surveillance. Additionally, the use of YOLOv5 for ripe tomato detection contributes to high accuracy along with real time detection. Consequently, the rover proves to be a versatile and valuable tool for smart agriculture with its multifunctional capabilities. The results demonstrate the rover’s capability to enhance crop management practices and optimize soil health, providing valuable insights for future advancements in precision agriculture. The findings of this study provide valuable insights for agricultural stakeholders and researchers seeking to optimize farming operations through advanced technologies.

## Supporting information

S1 File See Supplement 1 for supporting content. (DOCX)
