# Supplementary material for: Designing and development of agricultural rovers for vegetable harvesting and soil analysis
Source: PLoS One. 2024 Jun 21;19(6):e0304657. doi: 10.1371/journal.pone.0304657 (PMC11192377; doi:10.1371/journal.pone.0304657)
Supplement: S1 File — (DOCX) [file pone.0304657.s001.docx]

**Design and development of agricultural rovers for vegetable harvesting and soil analysis.**

Bristy Das^a^, Tahmid Zarif Ul Hoq Sayor^a^, Rubyat Jahan Nijhum^a^,Mehnaz Tabassum Tishun^a^,Taiyeb Hasan Sakib^a^, Md. Ehsanul Karim^a^, AFM Jamal Uddin^b^, Aparna Islam^c^, and Abu S. M. Mohsin^a*^

^a^ Nanotechnology, IoT and Applied Machine Learning Research Group, EEE Dept, Brac University, Dhaka, Bangladesh

**^b^**Department of Horticulture,Sher-e-Bangla Agricultural University (SAU), Dhaka,Bangladesh

**^c^**Biotechnology Program, Department of Mathematics and Natural Sciences,Brac University, Dhaka, Bangladesh  **Corresponding Author: ***Abu S. M. Mohsin**,** E-mail: [asm.mohsin@bracu.ac.bd](mailto:asm.mohsin@bracu.ac.bd)

**Supplementary S1**

**1. Performance Metrics**

$$Accuracy = \frac{TP + TN}{TP + FP +TN +FN}$$

$$Precision = \frac{TP}{TP+ FP}$$

$$Recall = \frac{TP}{TP + FN}$$

$$F1 Score = \frac{2*( Recall* Precision)}{Recall + Precision}$$

$$mAP = \frac{1}{N}\sum_{i =1}^{i = N} \left| Pi \right|$$

$$IoU = \frac{Area of Intersection}{Area of Union}$$

TP is True Positive, TN is True Negative, FP is False Positive and FN is False Negative. P_i_

The Mean Average Precision (mAP) is the precision at a certain threshold of Intersection-over-Union (IoU). The IoU is a measure of how much overlap there is between a predicted bounding box and the ground truth bounding box.

**3. F1 Curve:**

**
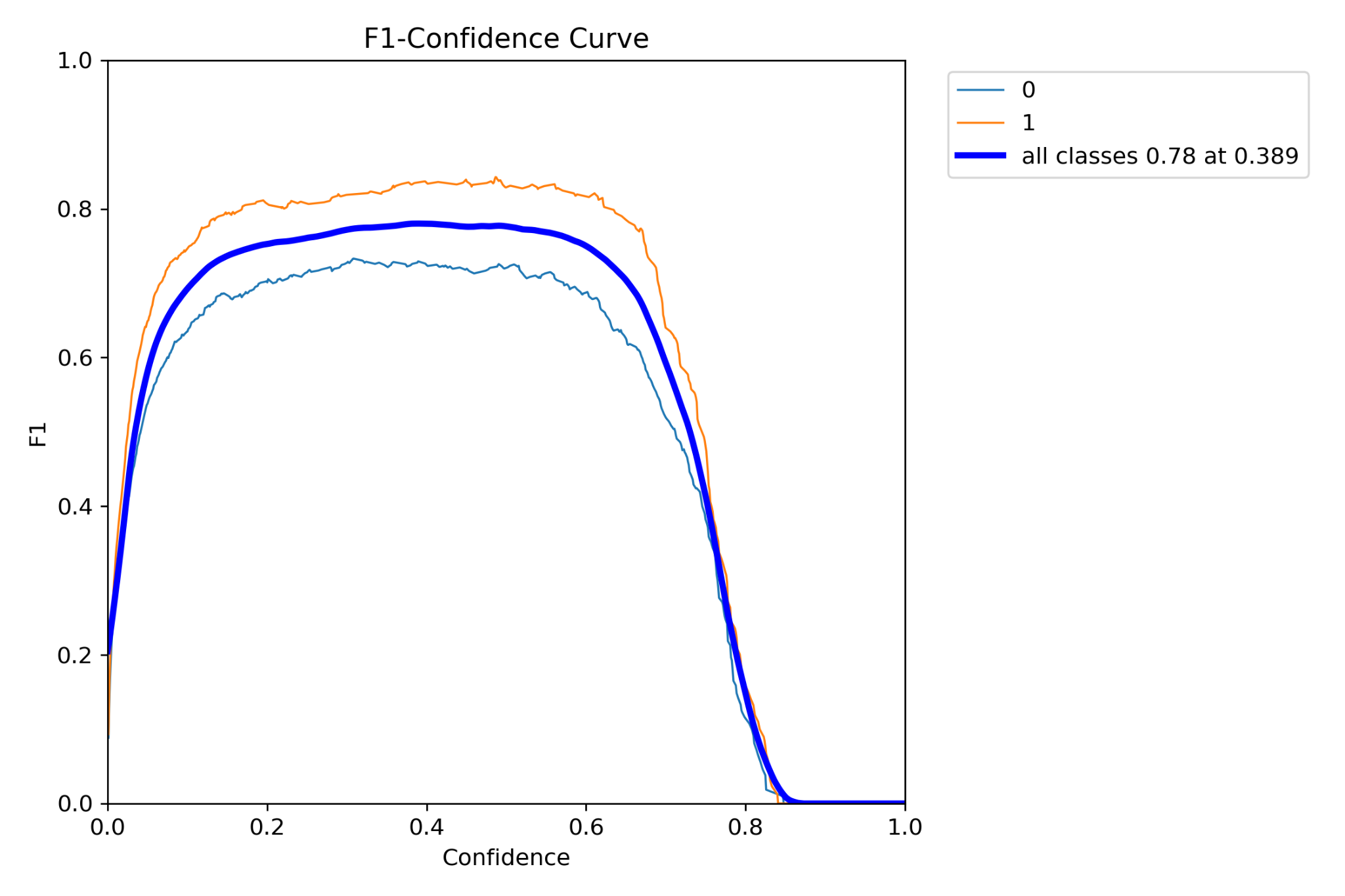
**

**Fig. S1.**  F1-Confidence Curve of trained YOLOv5. Each point on the line indicates the F1-score at the given confidence filter constant. The peak of the F1-score is 0.78, reached at the confidence of 0.389. The F1 is a good performance indicator considering both false negatives and positives.

**4. PR Curve:**

**
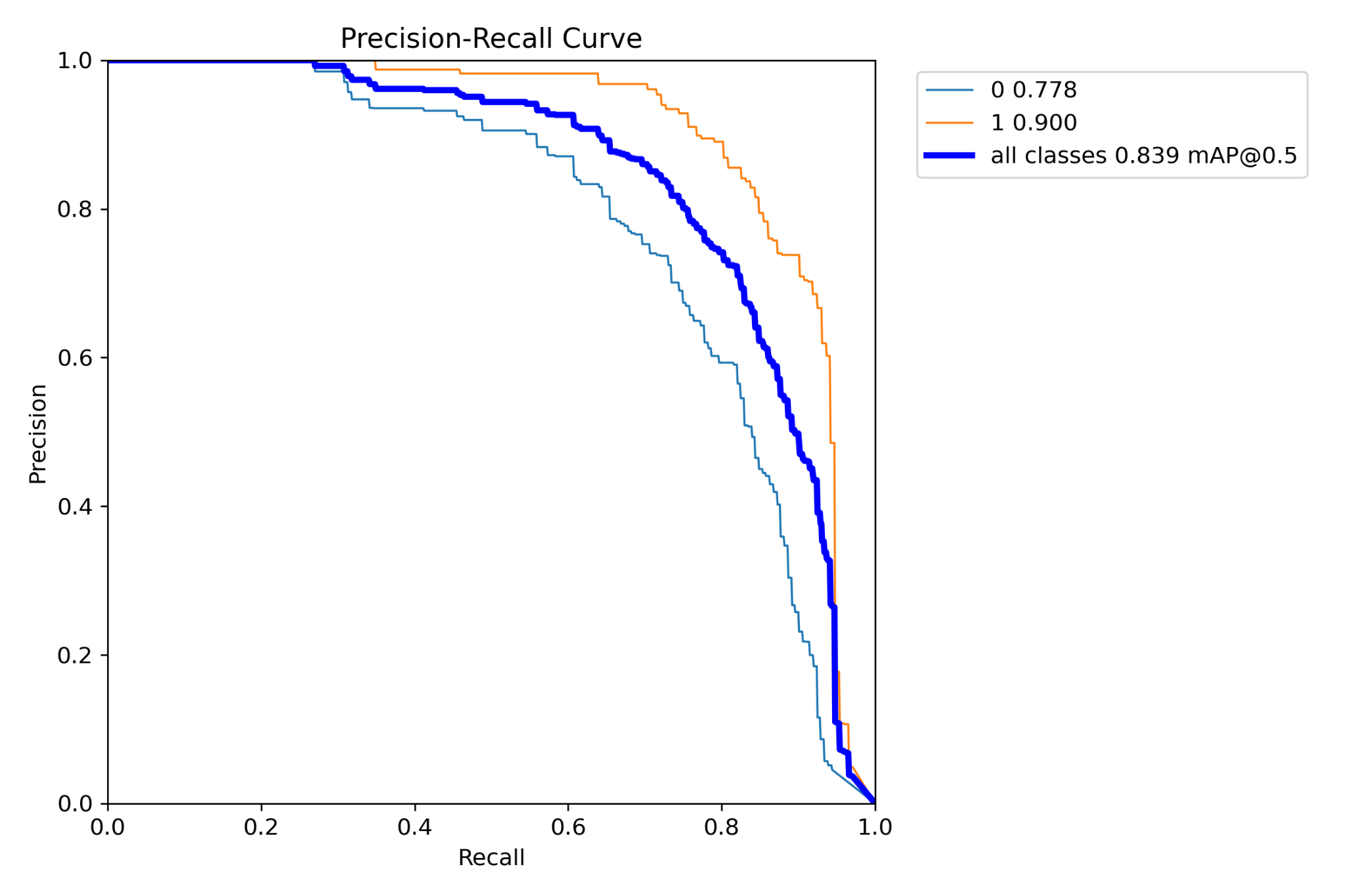
**

**Fig. S2.**  Precision-Recall Curve of trained YOLOv5. Each point on the line indicates the variation of Precision with Recall as the threshold value is varied.
